# Comparative Analysis of Bond Strength Durability of 10-Methacryloyloxydecyl Dihydrogen Phosphate-Containing Adhesives on a Low-Viscosity Bulk-Fill Composite Surface

**DOI:** 10.3290/j.jad.b3608775

**Published:** 2022-11-28

**Authors:** Renáta Martos, Melinda Szalóki, József Gáll, Attila Csík, Csaba Hegedűs

**Affiliations:** a Assistant Professor, Department of Operative Dentistry and Endodontics, Faculty of Dentistry, University of Debrecen, Debrecen, Hungary. Conceptualization, methodology, investigation, data curation, visualization, prepared original draft, reviewed and edited the manuscript, read and agreed to the published version .; b Assistant Professor, Department of Biomaterials and Prosthetic Dentistry, Faculty of Dentistry, University of Debrecen, Debrecen, Hungary. Methodology, investigation, data curation, visualization, prepared original draft, reviewed and edited the manuscript, read and approved the published version.; c Associate Professor, Department of Applied Mathematics and Probability Theory, Faculty of Informatics, University of Debrecen, Debrecen, Hungary. Formal analysis, prepared original draft, read and approved the published version.; d Senior Research Associate, Institute for Nuclear Research (ATOMKI), Debrecen, Hungary. Methodology, investigation, prepared original draft, read and approved the published version.; e Professor, Department of Biomaterials and Prosthetic Dentistry, Faculty of Dentistry, University of Debrecen, Debrecen, Hungary. Conceptualization, resources, supervision, project administration, funding acquisition, read and approved the published version.

**Keywords:** low-viscosity bulk fill composite, universal adhesives, self-etch adhesive, 10-MDP, aging, bond strength.

## Abstract

**Purpose:**

To compare the bond durability of adhesives with 10-methacryloyloxydecyl dihydrogen phosphate (10-MDP) to low-viscosity bulk-fill composite.

**Materials and Methods:**

Four 10-MDP-containing adhesives (Tokuyama Bond Force II [TBF II], Tokuyama; Scotchbond Universal [SU], 3M Oral Care; Clearfil Universal Bond Quick [CL], Kuraray Noritake; and G-Premio Bond [GP], GC) and one 10-MDP-free adhesive (Heliobond [HB] Ivoclar Vivadent) as a control were applied to polished, air-abraded surfaces of randomly assigned SureFil SDR flow low-viscosity bulk-fill composite blocks. The application of the adhesives was followed by applying Tetric EvoCeram universal nanohybrid composite in layers. Each layered composite block was sliced into stick specimens with a hard-tissue microtome. Half of the groups were randomly selected and tested for microtensile bond strength (immediate group); the other groups were aged in a thermocyling machine for 5000 cycles, followed by testing microtensile bond strength (aged group). The adhesive interface was evaluated with scanning electron microscopy (SEM). Failure modes were observed using light microscopy. The results were evaluated with Levene’s test, ANOVA, Welch’s ANOVA, Tukey’s test and the Z-test as appropriate (significance: p < 0.05).

**Results:**

There was a significant difference in the bond strength between 10-MDP-containing adhesives and the 10-MDP-free adhesive in all groups. Aging significantly decreased the bond strength in all adhesive groups. There was no significant difference in the bond strength durability among the 10-MDP-containing adhesives.

**Conclusion:**

Application of 10-MDP-containing adhesives has an advantageous effect on the air-abraded SDR composite surface compared with 10-MDP-free adhesive. The composition of 10-MDP-containing adhesives did not influence the bond strength. Aging diminishes the bond strength durability of 10-MDP-containing adhesives.

Because minimally invasive and esthetic approaches are favored in dentistry, light-cured resin composites (RBCs) are the first choice of restorative materials in daily clinical practice.^14^ Conventional composites should be layered incrementally during the restorative procedure, and the copolymerization of subsequent composite layers is usually enhanced by the oxygen-inhibiting layer (OIL) on the uppermost composite surface. As a simplification of the time-consuming and technically sensitive application protocol, bulk-fill resin composite (BFRC), which allows an increment thickness of 4-5 mm, was developed. BFRCs contain an alternative photoinitiator system and newly synthetised monomers associated with stress-decreasing technology.^27^ Flowable (low-viscosity) and full-body (high-viscosity) BFRCs are available, each with distinct clinical application sequences. However, low-viscosity BFRCs are mainly used as dentin-replacement materials, and a universal resin composite is required as a cap on top of the restoration.^27^

Certain clinical situations result in the loss or contamination of the OIL and may influence the application of a new composite layer. In these particular cases, the affected composite surface must be activated by roughening and/or wetting the surface to ensure adhesion between the composite layers.^18^ This method may be used as an immediate repair procedure. The key factor to achieve interface stability is the quality and durability of adhesion. Protocols highlight the importance of mechanical surface treatments and confirm the advantage of chemical conditioning methods,^13^ but conclusions about the ideal protocol do not all agree.^26^ Although the surface of fresh composite is quite favorable for activation compared with an aged, disintegrated composite surface, information about activation of a flowable bulk-fill composite surface with 10-methacryloyloxydecyl dihydrogen phosphate (10-MDP)-containing adhesives is scarce and the durability of the interface is also questionable.^26^

Universal adhesives are widely accepted due to their multimodal and multipurpose characteristics. Moreover, the application technique is simpler because they are self-etch adhesives. The advanced technology behind these adhesives provides etching, priming, and bonding in one step with reduced technical sensitivity.^28^ Universal and self-etch adhesives have a specific composition and complexity tailored to achieve stable and sufficient bond strength, but their water sorption and product-dependent efficacy are causes for concern. Universal and self-etch adhesives contain one or more acidic functional molecules that are responsible for enhancing conditioning and chemical interaction.^7^ 10-MDP is one of the most versatile functional monomers; it has an extremely high potential to adhere to various substrates, such as dental hard tissues, lithium disilicate, zirconia ceramics, and metals,^35^ and it seems to be a key factor for self-etch adhesives to achieve stable bond strength.^5^

The effectiveness of universal adhesives in different protocols has been investigated.^16^ Therefore, the purpose of this in-vitro study was to compare the effectiveness of four 10-MDP-containing adhesives on the low-viscosity bulk-fill composite surface and the reliability of the protocol after aging by determining the microtensile bond strength (µTBS). We used air abrasion as the gold-standard mechanical surface treatment^4^ combined with application of 10-MDP-containing adhesives and thermocycling for specimen aging (following ISO/TS 11405:2015).^9^ Three hypotheses were tested: 1. there is no significant difference between bond strengths of 10-MDP-containing adhesives with different compositions; 2. aging has no effect on the effectiveness of 10-MDP-containing adhesives; 3. there is no difference in the bond strength durability when comparing 10-MDP-containing adhesives with a 10-MDP-free adhesive.

## MATERIALS AND METHODS

### Study Materials

Five different adhesives – Heliobond (HB, Ivoclar Vivadent; Schaan, Liechtenstein), Tokuyama Bond Force II (TBF II, Tokuyama Dental; Tokyo, Japan), Scotchbond Universal (SU, 3M Oral Care; St Paul, MN USA), Clearfil Universal Bond Quick (CL, Kuraray Noritake; Tokyo, Japan) and G-Premio Bond (GP Bond, GC; Tokyo, Japan) – were applied on the surface of SureFil SDR Flow bulk-fill composite (Dentsply Sirona; Konstanz, Germany) as the substrate. Layering was completed with Tetric EvoCeram (TEC, Ivoclar Vivadent) universal nanohybrid composite. The description, composition and manufacturers’ details of the materials are listed in Table 1. All adhesives contained 10-MDP or its derivates, except HB, which served as a hydrophobic adhesive control, free of solvent and acidic monomers.

**Table 1:** Composition and manufacturers of resin composite and adhesive materials

Code	Material	Manufacturer	Components	Lot
SDR	SureFil SDR Flow bulk-fill composite	Dentsply Sirona; Konstanz, Germany	SDR patented UDMA, TEG-DMA, bis-EMA, CQ, BHT, UV stabilizer, TiO_2_ Ba-Al-F-B-silicate glass 68 wt% (44 vol%) nanofiller and Sr-Al-F-silicate glass 4.2 µm	1806000584
TEC	Tetric EvoCeram nanohybrid composite	Ivoclar Vivadent; Schaan, Liechtenstein	Bis-GMA, UDMA, bis-EMA, CQ, Lucirin TPO, stabilizers, B-Al-silicate glass fillers, YbF_3_, macrofiller of mixed oxides	X51351
HB	Heliobond adhesive	Ivoclar Vivadent	Bis-GMA 59.5 wt%, TEG-DMA 39.7 wt%, CQ Stabilizers and catalysts 0.8 wt%	X10508
TBF II	Tokuyama Bond Force II adhesive	Tokuyama Dental; Tokyo, Japan	3D-SR phosphate monomer*, HEMA, bis-GMA, TEG-DMA, water, alcohol, CQ, catalyst	097
SU	Scotchbond Universal adhesive	3M Oral Care; St Paul, MN, USA	10-MDP phosphate monomer, bis-GMA, DCDMA, EDMAB, MPTMS, DMAEMA, VCP, HEMA, ethanol, water, CQ, treated silica	80409A
CL	Clearfil Universal Bond Quick adhesive	Kuraray Noritake; Tokyo, Japan	10-MDP, bis-GMA, HEMA, hydrophilic amid methacrylate, MPTMS, NaF, colloidal silica, sodium-fluoride, CQ, ethanol, water	3K0206
GP	G-Premio Bond adhesive	GC; Tokyo, Japan	4-MET, 10-MDP, MTDP, methacrylic acid ester, silica, catalyst, photoinitiators, acetone, water	1906121 012687

Bis-GMA: bisphenol A-diglycidyl dimethacrylate; EDMAB: ethyl-4-(dimethylamino)benzoate; UDMA: urethane dimethacrylate; bis-EMA: ethoxylated bisphenol A dimethacrylate; TEG-DMA: triethylene glycol dimethacrylate; DMAEMA: 2-(dimethyl amino)ethyl methacrylate; HEMA: 2-hydroxyethyl methacrylate; CQ: camphorquinone; Lucirin TPO: 2;4;6 trimethylbenzoyldiphenylphosphine oxide; 10-MDP: 10-methacryloyloxydecyl dihydrogen phosphate; TiO_2_: titanium-dioxide; YbF_3_: ytterbium trifluoride; BHT: butylated hydroxytoluene; VCP: Vitrebond copolymer (copolymer of acrylic and itaconic acid); DCDMA 1; 10-decamethylene dimethacrylate; MPTMS: γ-methacryloxypropyl trimethoxysilane; NaF: sodium fluoride. *3D-SR: three-dimensional self-reinforcing monomer (a modified MDP molecule).

### Specimen Preparation for µTBS Measurements

**Fig 1:**
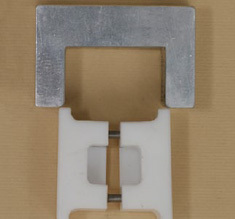
Custom- made Teflon mold.

SDR blocks were prepared in a 10 mm x 10 mm x 7 mm custom-made Teflon mold. Four-millimeter-thick layers were applied according to the bulk-fill technique (Fig 1). Each increment was polymerized for 180 s in a Scheu LC-6 light oven (Iserlohn, Germany) equipped with different light tubes (three UVA, three blue light, with maxima of 370 nm and 450 nm, respectively).

#### Surface treatment of SDR blocks

The adhesive surface of the SDR blocks were polished with 500-, 1000- and 1200-grit silicon-carbide abrasive papers under water cooling using a polishing machine (Struers LaboPol35; Rødovre, Denmark) at 300 rpm for 30 s. After polishing, the blocks were cleaned in an ultrasonic bath for 10 min to eliminate the abrasive particles from the surface. The polished SDR blocks were sandblasted with 50-μm Al_2_O_3_ (Danville Engineering; San Ramon, CA, USA) using an intraoral sandblaster (Microetcher, Danville Engineering) from a distance of 10 mm at a pressure of 2.5 bar for 10 s, followed by washing (60 s) and drying (60 s) with an air-water syringe. The cured and polished blocks were kept dry at room temperature for 24 h before being adhesively bonded to TEC.

#### Application of adhesives (Fig 1)

After 24 h, using a disposable applicator, a thin coating of each adhesive was applied to a randomly chosen sandblasted SDR surface following the manufacturers’ instructions. The application modes of adhesives are summarized in Table 2. The adhesives were dried with an oil-free air-water syringe. All adhesives were light cured with a dental light-curing device (Bluephase 20i, Ivoclar Vivadent) set at a high-mode curing program (1200 mW/cm^2^).

**Table 2:** Application mode of adhesives

	Heliobond	Tokuyama Bond Force II	Scotchbond Universal	Clearfil Universal Bond Quick	G-Premio Bond
Duration of application (s)	Brushing motion	10 s	20 s	Cover the surface	Shake before use, cover the surface
Motion		Active; circular rubbing	Active; circular rubbing	Active rubbing motion	Active circular rubbing (wait 10 s before drying)
Drying time	5 s	5 s	5 s	5 s light pressure	5 s maximum air pressure
Polymerization time	10 s	20 s	10 s	10 s	10 s


#### Application of universal composite

After applying adhesives, the SDR blocks were put back in the Teflon mold and TEC composite repair was prepared according to the manufacturer’s instructions. The TEC composite was applied in 2-mm increments, and each layer was polymerized for 3 min in a Scheu LC-6 light oven. After 24 h, the repaired block was sliced in two directions with a diamond-saw–equipped hard-tissue microtome (Leitz 1600; Wetzlar, Germany) under water cooling. This produced 1 x 1 x 14 mm stick-shaped specimens. Thirty out of 90 non-trimmed sticks of each group were randomly selected and divided into two groups. The first was group submitted to µTBS measurements and the second to aging (see below).

#### Aging of the interface

**Fig 2:**
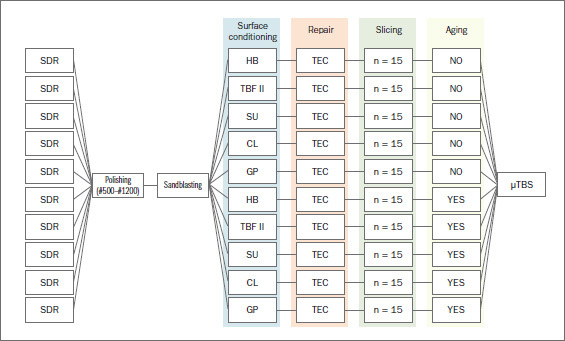
Flow chart of test groups. SDR: Smart Dentin Replacement composite; HB: Heliobond; TBF II: Tokuyama Bond Force II; SU: Scotchbond Universal; CL: Clearfil Universal Bond Quick; GP: G-Premio Bond; µTBS: microtensile bond strength measurements.

The second group of sliced specimens (1 mm x 1 mm x 14 mm) were aged in a thermocycling machine (THE-1100, SD Mechatronik; Feldkirchen-Westerham, Germany) for 5000 cycles at 5–55ºC with a 30-s dwell time. After aging, the µTBS of the sticks was measured. Figure 2 shows the experimental groups based on the applied adhesives and aging protocol.

### µTBS Measurements

The width and thickness of each specimen was measured at three different points with a digital calliper; these values were used to calculate the average width and thickness. The aged and non-aged sticks were attached to an active-grip notched metallic cuvette. The cuvette was placed into a mechanical analyzer (Instron 5544; Norwood, MA, USA) equipped with a 2-kN load cell. The crosshead speed was set at 1 mm/min. The µTBS was calculated by dividing the detected load (N) by cross-sectional area (mm^2^).

### Detection of the Failure Mode

All fractured surfaces were analyzed under a stereo light- microscope (Olympus SZ61, Olympus; Tokyo, Japan) at 45X magnification to determine the type of failure. The failures were divided into two groups: 1. adhesive, when the failure occurred at the interface between the SDR and TEC composite, and 2. cohesive, when the failure occurred in the SDR or TEC composite.

### Scanning Electron Microscopy (SEM)

The surface morphology of the samples was investigated with a dual-beam focused ion-beam Scios 2 scanning electron microscope (Thermo Fisher Scientific; Waltham, MA, USA) operated at a low accelerating voltage (2 keV). Low energy and short working distance (2 mm) were applied to study the surface morphology of insulating samples (eg, biological samples, aerogels, plastics, etc) without a gold layer coating. A special detector, a so-called in-lens detection system, has the ability to separate and collect secondary electrons, backscattered electrons, or a mixture of both types of signals. The advantage of the gold sputter-coating-free method is that gaps and discontinuities are not masked.

### Statistical Analysis

The homogeneity of variance was checked with Levene’s test. Then, for data with homogeneous variance, the means between groups were compared with one-way ANOVA. For data without homogeneous variance, the means between groups were compared using Welch’s ANOVA. We then used the appropriate post-hoc test, Tukey’s honestly significant difference (HSD) test or the Tamhane test, for pairwise comparisons. Binomial tests were applied to determine adhesive or cohesive proportions that were different from 50%. To compare the proportions of adhesive fractures between immediate and aged cases, a two-sample Z-test for proportions was employed. SPSS Statistics 27 (IBM; Armonk, NY, USA) was used for all tests except the two-sample Z-test of proportions, which was calculated in R.^20^

## RESULTS

### µTBS Results

**Fig 3:**
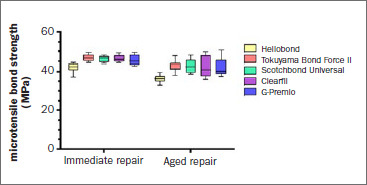
Microtensile bond strength results according to the tested adhesives and aging protocol (box plots are shown).

The µTBS data are shown in Fig 3. The mean µTBS of the tested protocols was between 36.4 ± 2.0 MPa and 46.6 ± 1.6 MPa. There was a significant difference in µTBS between the 10-MDP-containing adhesives and the 10-MDP-free adhesive in all groups (p < 0.05). Aging significantly reduced µTBS in all adhesive groups (p < 0.05). There were significantly higher variances in µTBS in the aged groups of 10-MDP-containing adhesives (p < 0.05), which was associated with wider ranges and lower minima (Fig 3). For the 10-MDP-containing adhesives, µTBS did not differ significantly between the immediate and aged groups (p ˃ 0.05).

### Failure Mode Analysis

**Fig 4:**
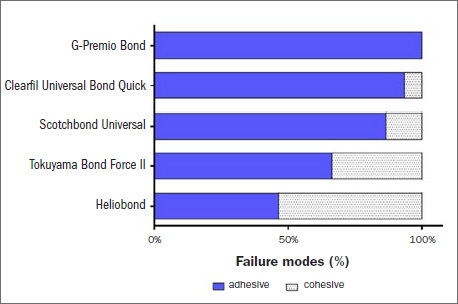
Immediate repair failure modes of the tested adhesives.

**Fig 5:**
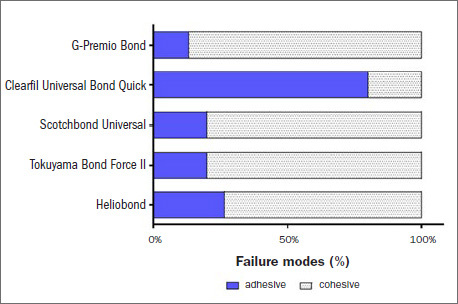
Aged repair failure modes of the tested adhesives.

Figures 4 and 5 show failure mode results. Light microscopic analysis revealed a significantly higher rate of adhesive failure in the immediate groups with 10-MDP-containing adhesive: 100% for GP Bond, 93.3% for CL, 86.7% for SU and 66.7% for TBF II. However, the dominant failure mode for HB was cohesive failure (53.3%). For the most part, the aged groups presented significantly greater percentages of cohesive failure: 86.7% for GP bond, 80% for SU, 80% for TBF II and 73.3% for HB. In contrast, adhesive failures dominated in the aged CL group (80%).

### SEM

**Fig 6:**
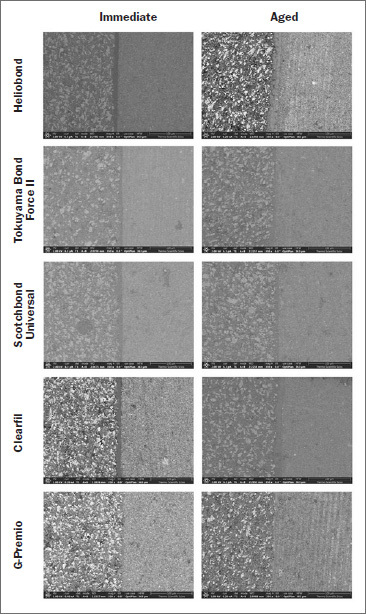
SEM images of the repaired interfaces according to the applied adhesives and aging protocol (left half of each image: SDR; right half of each image: TEC).

Figure 6 shows the SEM results according to the applied adhesives and aging protocol. SDR is shown in the left half of each image, and TEC is shown in the right half of each image. The scanning electron micrographs show inhomogeneous size distribution of large filler particles in the SDR composite, while the TEC has a smoother surface. There are well-defined interfaces in both the immediate and aged samples.

## DISCUSSION

In this study, we evaluated the µTBS of four 10-MDP-containing adhesives (Tokuyama Bond Force II [TBF II], Scotchbond Universal [SU], Clearfil Universal Bond Quick [CL], and G-Premio Bond [GP]) to a low-viscosity bulk-fill resin composite and examined the bond strength before and after a thermocycling regimen. Self-etch and universal adhesives were launched to overcome the drawbacks of multistep etch-and-rinse adhesives used for definitive restorations and to provide chemical adhesion in certain clinical situations.^16,30^ The chemical reactions provide great improvement in the quality of adhesion and highly influence the chemical composition of self-etch adhesives. Therefore, the clinical performance and efficacy of self-etch adhesives are influenced by interactions of the different adhesive components, application protocols, and surface quality of the substrate.^15,31,32^ Simplified adhesives contain a mixture of hydrophilic and hydrophobic molecules, but the purity and the concentration of the monomers vary according to the different products, which strongly affects their bond strength and durability.^8^

Although the long-term effectiveness of universal adhesives on dentin and enamel has been investigated previously,^2,15,25,39^ data regarding the bond strength of 10-MDP-containing adhesives on flowable bulk-fill composite surfaces is scarce. The universal and self-etch adhesives containing 10-MDP tested in this study can bond to a variety of substrates, including zirconia, metal oxides, silica, and resin monomers.^35^ The connecting molecules may establish a stable nanolayered structure as a protective zone against biodegradation, associated with enhanced bonding performance at the adhesive interface.^5^

HB is an adhesive free of acidic functional monomer; hence, we used it as a control bonding agent. Adhesives with acidic functional monomers contain organic solvents (alcohol or acetone) that reduce the viscosity of the monomer mixture and help the monomers to penetrate surface irregularities. After drying the adhesive layer, solvent can be entrapped in the interfacial layer because of the good miscibility of the solvent and the monomers. The remaining solvent can influence the adhesion between SDR and TEC. This phenomenon is not observed with the solvent-free HB adhesive. A slightly viscous monomer mixture serves to fill the irregularities of the sandblasted composite surface based on their molecular mobility.

We used SDR bulk-fill resin composite as a substrate for the tested adhesives. SDR demonstrates a high degree of conversion and had a moderate filler load with barium-aluminium-silica-based particles of various sizes (800–4200 nm).^12^ These large particles can be advantageous as a retentive area for resin bonding agents.^3^ Adhesion at the composite-composite interface is influenced by both mechanical factors and chemical components.^13,26^ Therefore, prior to adhesive application, the composite resin surfaces were ground with silicon carbide disks (up to 1200 grit) followed by air abrasion with 50-µm Al_2_O_3_ particles. This protocol is relevant to clinical situations in which finishing an RBC restoration is followed by an immediate correction due to failure. Fresh composite represents an idealised surface, free of signs of hydrolysis or degradation. Unreacted monomers provide C=C to form C-C covalent bonds with the intermediate agent. Moreover, functional monomers connect with the fillers so that the bond strength may increase the cohesive strength of the composite substrate.^3^

The µTBS of the 10-MDP-containing adhesives investigated here are in line with data reported by Ahmed et al,^2^ Yilmaz et al,^34^ and Sismanoglu et al.^22^ Those authors found effective bonding to the flowable bulk-fill composite, which is consistent with our SEM results. Furthermore, the µTBS of all tested 10-MDP-containing adhesives was significantly higher than with the control adhesive in the immediate groups, which is consistent with the results of a previous study.^10^ The difference in the composition of the 10-MDP-containing adhesives in this study did not result in significantly different µTBS, similar to findings by Isolan et al^11^ and Suarez et al,^6^ but in contrast to another study.^22^ Therefore, we accepted our first hypothesis. We hypothesised that applying self-etch adhesives with a rubbing motion promotes infiltration into the air-abraded composite surface, a phenomenon associated with stronger bonds.^15^ During application, GP-Premio Bond required a short burst of maximum air pressure, which is consistent with the thinner GP-Premio Bond adhesive layer observed in scanning electron micrographs. The air-thinning step of the application protocol may affect the bond layer thickness, but it hardly seems affected by the presence of the filler component, as seen in the scanning electron micrographs.

Silanization as a separate priming step prior to adhesive application has been suggested to improve wetting,^26^ and improves µTBS. Researchers have hypothesized that including silane in adhesives improves the wetting and the bonding ability^24^ similar to a separate silanization step.^10^ Incorporation of silane into the adhesive agent may simplify the protocol, but the beneficial effect on µTBS may also be influenced by the composition and pH of the bonding agent.^38^ Regarding the silane content, adhesives with silane (SU and CL) or without silane (TBF II and GB) showed similar µTBS in the immediate and aged groups. These findings are in agreement with Moritake et al,^15^ Suzuki et al,^25^ and Ouchi et al.^17^ Due to the acidic pH of SU and CL, the stability of silane may be compromised, resulting in a modified chemical formula with a lower priming capacity.^38^ This phenomenon could account for the quite similar µTBS of universal and self-etch adhesives with and without silane.

As a component of dental adhesives, 2-hydroxyethyl methacrylate (HEMA) may act partially as a solvent to prevent phase separation and may improve surface wetting. However, it has also been associated with high water uptake^29^ and an inhibitory effect on polymerization and formation of the 10-MDP interfacial nanolayer.^36^ Of the adhesives we tested, only GP-Premio Bond is HEMA free, but GP-Premio Bond did not have a significantly higher µTBS compared with the other 10-MDP-containing adhesives. This finding is different from the results obtained by Ahmed et al.^1^

SU contains Vitrebond copolymer (VCP, a patented form of polyalkenoic acid copolymer) that is based on self-adhesive glass-ionomer technology; it has shown superior bonding performance.^23^ In this study, SU did not increase the repair bond strength compared with the other universal or self-etch adhesives, in agreement with a previous study.^2^ A possible explanation could be that the interactions between components of SU, such as the high-molecular-weight polyalkenoic copolymer, may impair adhesion of 10-MDP to the same substrate.^37^ Furthermore, the resin components may impede the polyalkenoate reaction.^21^

Thermocycling is a suitable method for simulating the effects of hydrolysis, water sorption, and thermal stress; thus, it is appropriate for testing the durability of the bonded interface. Hydrolysis of the resin polymer and resin-filler interface, monomer leaching, degradation of the cross-linked matrix, microcrack formation, and deterioration of the bonded resin interface reduce the repair bond strength.^3,25,33^ In agreement with Moritake et al,^15^ Altinci et al,^3^ and Zhang et al,^39^ but in contrast with other bond-strength durability results,^25^ in our study the bond strength was significantly lower in the aged than in the immediate groups. Hence, we rejected our second hypothesis. This outcome is consistent with the limited hydrolytic stability of self-etch adhesives. Hydrophilic components with hydroxyl or phosphate groups, HEMA, or silane may accelerate the deterioration of the bonded interface.^19^ Despite this phenomenon, the µTBS of the 10-MDP-containing adhesive groups was significantly higher than that obtained with HB. While the hydrophobic resin layer has been suggested to serve as a protective layer to reduce hydrophilic degradation of universal adhesives,^1^ the reduction in µTBS of HB was also significant. The µTBS reduction was 9% for TBF II, 9% for SU, 10% for CL, 8% for GP, and 13% for HB. These changes demonstrate a similar degradation tendency in all adhesive groups, independent of the composition or application protocol on the bulk-fill resin composite surface. Considering the hydrophobic nature of 10-MDP, the chemical interaction between functional monomers and the SDR composite surface may decrease the deterioration of the adhesive interface.^30^ Therefore, we rejected our third hypothesis.

Regarding the fracture types in the immediate groups, there was a higher percentage of adhesive fractures, except for HB, indicating the similarities of the 10-MDP-containing adhesives. The cohesive fracture type was the main type detected after aging, in accordance with Altinci et al^3^ and Moritake et al,^15^ except for CL. This difference could be associated with the presence of the hydrophilic amide methacrylate component. The dominance of cohesive fractures may be associated with the hydrolytic degradation and softening of the resin matrix, and loosening of the filler particles in parallel with interface disintegration,^3,26^ although the scanning electron micrographs showed gap-free, well-integrated, tight interfaces in all groups.

Our use of µTBS to evaluate bond strength is consistent with previous studies.^6,26^ However, in-vitro studies have well-known limitations. Thus, additional studies should evaluate the effect of longer aging or the bond strength durability of multiple-layered adhesives.

## CONCLUSIONS

The composition of 10-MDP-containing adhesives does not influence the µTBS to a low-viscosity bulk-fill resin composite. Aging has a deteriorating effect on the bond strength of 10-MDP-containing and 10-MDP-free adhesives. After aging, 10-MDP-containing adhesives seem to be more effective and durable than the non-solvated, 10-MDP-free adhesive at the SDR-TEC interface.

## Clinical relevance:

Compared with 10-MDP-free adhesive, all the tested 10-MDP-containing adhesives resulted in acceptable bond strength to a low-viscosity bulk-fill substrate. Therefore, any of these adhesives could be chosen for low-viscosity bulk-fill resin composite activation.
